# Multiplanar Reconstructed Thoracic CT Bronchoscopy in Endobronchial Tuberculosis

**DOI:** 10.5812/iranjradiol.8726

**Published:** 2012-11-20

**Authors:** Fariba Rezaeetalab, Donya Farrokh, Behrouz Zandiee

**Affiliations:** 1 Department of Pulmonary Medicine, Lung Diseases and TB Research Center, Imam Reza Hospital, Mashhad University of Medical Sciences, Mashhad, Iran; 2 Department of Radiology, Imam Reza Hospital, Mashhad University of Medical Sciences, Mashhad, Iran

**Keywords:** Tomography, Spiral Computed, Tuberculosis, Bronchial

Dear Editor,

Pulmonary tuberculosis is one of the most important health problems in the world (1, 2). Human immunodeficiency virus (HIV) infection, immigration, multidrug resistance, delay and failure in prevention, diagnosis and treatment cause a high incidence of pulmonary tubrculosis (3). Endobronchial tuberculosis (ETBT) is a tracheobronchial inflammatory disease caused by tuberculosis bacillus. EBTB has at least three important findings. First, EBTB is a highly infectious and contagious infectious disease. Second, the clinical and radiological features are not specific and misleading causing delay in the definite diagnosis. Third, EBTB causes sequels such as airway stenosis (4). On the other hand, in EBTB, the incidence of positive sputum smear and culture for Bacille de Koch (BK) may be low. Therefore, a negative sputum sample for TB does not exclude the diagnosis. The gold standard method for diagnosis is invasive fiberoptic bronchoscopy with bronchoscopic samplings (including bronchial washing for smear and culture with tissue biopsy) (2, 4). Clear and normal chest radiography does not rule out EBTB (4, 5). CT scan is more sensitive than chest- x ray in the diagnosis of early endobronchial involvement (5). Multiplanar reconstructed thoracic CT has been employed for noninvasive evaluation of endobronchial lesions. So, multiplanar reconstructed thoracic CT may improve confidence in diagnosis over axial imaging alone (6, 7). Reconstructed images in the coronal plane were carried out for forty patients with endobronchial tuberculosis, by multiplanar reconstructed thoracic CT in Imam-Reza hospital, Mashhad, Iran from 2009 to 2011. All of them underwent chest X-ray and fiberoptic bronchoscopy. The patients’ bronchial smear and culture for BK were positive. In addition, tissue from the endobronchial lesion showed granuloma with caseating necrosis. The mean age was 52.8 ± 19.26 (13 to 87) years; 23 (57.5%) were female and 17 (42.5%) were male. The major symptoms were cough, sputum, hemoptysis, fever, weight loss and night sweating (Table 1).

**Table 1:** Symptoms and Clinical Findings in Patients with Endobronchial Tuberculosis

Clinical Finding	No., (%)
**Symptoms**	
Cough	36 (90)
Sputum	35 (87.5)
Hemoptysis	8 (20)
Fever	34 (85)
Night Sweats	21 (52.5)
Loss of Weight	28 (70)
Chest Pain	5 (12.5)
Shortness of Breath	6 (15)
**Sounds**	
Crackle	7 (17.5)
Ronchi	4 (10)
Localized Wheezing	12 (30)
Generalized Wheezing	2 (5)
Bronchial Sound	9 (22.5)

**Figure 1:**
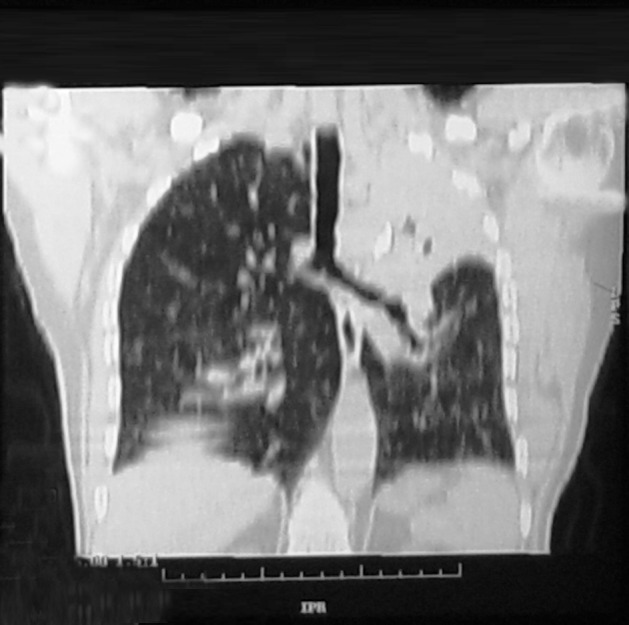
Endobronchial tuberculosis in multiplanar reconstructed thoracic bronchoscopy (stricture and narrowing in the left main bronchus)

In chest radiography, total or partial collapse of the lobes with volume loss were presented in 40%, consolidation with collapse was detected in 42.5 %, mass in 7.5%, cavitary lesion in 5%, extensive involvement with destructive lung in 3.5%. Normal chest -X rays were seen in 3% of the patients. The macroscopic appearance of lesions in fiberoptic bronchoscopy was inflammation, reddened and swollen mucosa with irregularity in 37.5%, ulcer in 17.5%, and stricture in 45% and mass in 7.5%. Multiplanar reconstructed thoracic CT bronchoscopy showed irregularity in 50% (Figure 1), mass in 7.5%, stricture in 42.5% and cavitary lesion in 5%.

One-hundred percent of endobronchial tuberculosis had abnormality in multiplanar reconstructed thoracic CT bronchoscopy and fiberoptic bronchoscopy (P = 0.217). CT scan can be used for noninvasive evaluation of the airways and surrounding structures. multiplanar reconstructed thoracic CT bronchoscopy is a novel technical image that reconstructs the intraluminal space and extra luminal tissues of the airways (7). In addition, the recent automated reconstruction can help evaluate the craniocaudal extent of an airway abnormality and stenosis, and determine abnormal anatomical findings before fiberoptic bronchoscopy (6). Multiplanar reconstructed thoracic bronchoscopy simulates a bronchoscopist's view of the airways. This noninvasive radiologic method generates endoluminal airway imaging (7, 8). On the other hand, multiplanar reconstructed thoracic bronchoscopy may avoid the inherent risks of invasive procedures such as conventional bronchoscopy in critically ill patients .Thus, we nominate that the results of fiberoptic bronchoscopy were nearly similar to multiplanar reconstructed thoracic CT bronchoscopy.
